# Interoperable chemical structure search service

**DOI:** 10.1186/s13321-019-0367-2

**Published:** 2019-06-28

**Authors:** Miroslav Kratochvíl, Jiří Vondrášek, Jakub Galgonek

**Affiliations:** 1 Institute of Organic Chemistry and Biochemistry of the CAS, Flemingovo náměstí 2, 166 10 Prague 6, Czech Republic; 2 Department of Software Engineering, Faculty of Mathematics and Physics, Charles University, Malostranské náměstí 25, 118 00 Prague 1, Czech Republic

**Keywords:** Substructure search, Small molecule databases, Interoperability, Linked data

## Abstract

**Motivation:**

The existing connections between large databases of chemicals, proteins, metabolites and assays offer valuable resources for research in fields ranging from drug design to metabolomics. Transparent search across multiple databases provides a way to efficiently utilize these resources. To simplify such searches, many databases have adopted semantic technologies that allow interoperable querying of the datasets using SPARQL query language. However, the interoperable interfaces of the chemical databases still lack the functionality of structure-driven chemical search, which is a fundamental method of data discovery in the chemical search space.

**Results:**

We present a SPARQL service that augments existing semantic services by making interoperable substructure and similarity searches in small-molecule databases possible. The service thus offers new possibilities for querying interoperable databases, and simplifies writing of heterogeneous queries that include chemical-structure search terms.

**Availability:**

The service is freely available and accessible using a standard SPARQL endpoint interface. The service documentation and user-oriented demonstration interfaces that allow quick explorative querying of datasets are available at https://idsm.elixir-czech.cz.

## Introduction

The vast availability of research-related data sources on the Internet has created a need for tools that can efficiently search through these data and automatically collect and associate information from multiple interoperable sources. In the chemistry field, chemical structure search is an extremely useful tool provided by many small-molecule databases as a free, on-line service. However, such services typically offer a simple, human-oriented user interface, which makes them unsuitable for automated use (e. g. by other services) or use in arbitrary combinations with other interoperable data sources. Here, we introduce a service that uses a standard-compliant interoperable interface to address these deficiencies and provide high-performance chemical structure search across many available chemical databases.

To promote interoperability among the diverse data sources accessible on the Internet, the World Wide Web Consortium (W3C) introduced semantic web technologies [1, 2]. The properties of semantic technologies make them ideal for interoperable publishing of datasets, the use of which has been consistently increasing over the past decade. Semantic data structured according to the *Resource Description Framework* (RDF) [3] are now routinely published by many well-established databases, including those from EBI, EMBL, UniProt, neXtProt, and KEGG. Interoperability of datasets provides the ability to automatically answer complex heterogeneous queries that cannot be answered by individual databases separately. However, the ability to pose chemical structure-driven queries over interoperable databases is currently limited. Consequently, comprehensive information about structural relations is largely absent in chemistry-oriented RDF datasets. This also limits opportunities to observe relationships with other linked information, such as proteins and metabolic pathways that are connected by chemically similar ligands or metabolites. To address this issue, we developed a publicly available service that augments the interoperable chemical search space by providing chemical similarity and substructure relations. This creates new ways to query and obtain more precise, meaningful data from currently available data sources.

Database interoperability is made possible by widespread standardization of data models and interfaces, and RDF is the key standardized semantic technology for interoperable representation of datasets [4]. Individual entities in RDF-formatted datasets are identified by *Internationalized Resource Identifiers* (IRI), which guarantee single global identification of each entity and avoid potential collisions of identifiers across datasets. *RDF vocabularies* are published to globally standardize identifiers for particular use cases [5, 6] to make data more discoverable and prevent ambiguity in interpretation. Query services for published RDF datasets typically support the *SPARQL* query language [7], which can be used to query datasets online, without the need to download them. Each SPARQL-supporting service is identified by a unique IRI, called a SPARQL *endpoint* [8].

Importantly, many SPARQL services support the *Federated Query Extension* [9]. With this extension, the user may instruct a service to execute specified parts of the query on other services and combine the collected data in one federated result [10]. This virtually removes the boundaries between different data sources, which greatly simplifies heterogeneous querying of the available data and promotes interoperability.

RDF specifies that datasets be modeled as sets of RDF *triples* [4], which provides sufficient expressive power to describe any data that can be modeled as graphs, including similarity and substructure relations between molecules. Current implementations of RDF databases, on the other hand, are typically only able to handle finite amounts of statically stored RDF entries, which leads to technical problems when handling data that may potentially match large numbers of RDF triplets. Chemical similarity and substructure relations are common examples of such data. Storing a predicate like hasSimilarity for each pair of molecules in a database is not only impractical, but technically impossible if the predicate should match structures not present in the databases (e. g. arbitrary structures defined by SMILES).

In classical SQL-based relational databases, this issue can be easily solved, for example, by implementing a stored procedure that dynamically creates the relations for the given parameters. Unfortunately, there is no similar concept standardized for RDF and SPARQL. We address this issue for the specific case of chemical search—the presented service is a publicly available SPARQL server that provides similarity and substructure relations generated on demand. Additionally, the procedure calls do not require extension of the SPARQL syntax, as they are triggered by simple data patterns in queries. Thus, the extension is transparent to services that are not aware of the involved procedure calls, which ensures compatibility with existing interoperable services.

A running instance of the chemical structure search service is available for free use at https://idsm.elixir-czech.cz/, as a part of the larger *Integrated Database of Small Molecules* (IDSM) project.

## Implementation

Overall, the service implementation can be viewed as an interoperable wrap of the structure search interface of the Sachem cartridge [11].

Sachem is an open-source chemical cartridge that we previously designed to provide chemical search capabilities in small-molecule databases, with performance sufficient for online services. Sachem exposes its search functionality using SQL stored procedures provided in a special PostgreSQL extension, which handles the necessary low-level, high-performance chemical indexing and search. Using the available SQL functions, Sachem users may create indexes of large searchable datasets and run substructure and similarity searches on them.

The main improvement offered by the new service is the ability to make the search interfaces of a running Sachem installation publicly available and interoperable, thus allowing anyone to quickly search through the indexed datasets. This functionality is provided by a generic SPARQL-to-SQL translator, which is the main part of the IDSM SPARQL engine.

**Fig. 1:**
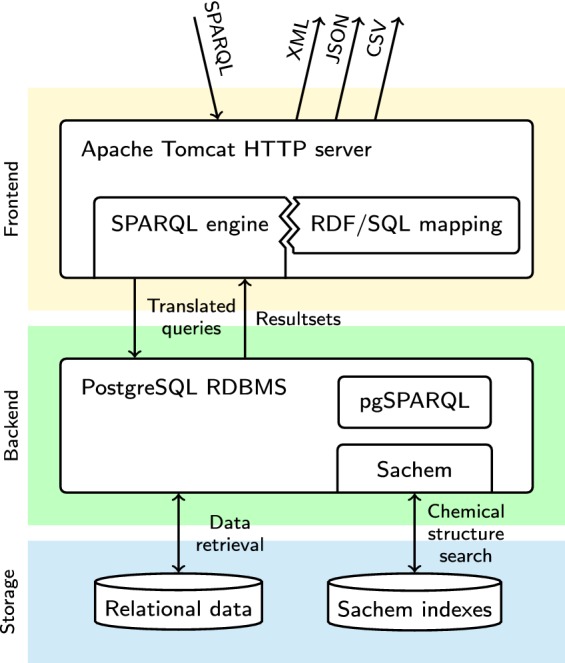
Overview of the interoperable chemical structure search implementation, separated into logical layers. Clients submit the SPARQL queries to the frontend HTTP server in Apache Tomcat. The incoming queries are passed to the SPARQL engine, which uses the connected RDF/SQL mapping to translate them into equivalent SQL queries. The resulting queries are evaluated by backend PostgreSQL DBMS, using the Sachem extension (which executes chemical structure search) and pgSPARQL extension (which evaluates SPARQL-specific parts of the query and gathers metadata). Stored data include the relational data in PostgreSQL and specialized chemical indexes in Sachem. Resultsets of the evaluated queries are passed back to the SPARQL engine, translated into a SPARQL result in the requested format, and sent back to the client

All components of the service are displayed in the overview in Fig. 1.

### IDSM SPARQL engine

Through our implementation of a SPARQL engine, we aimed to address several shortcomings of other RDF triple stores and SPARQL engines. Our implementation bridges the gap between triplet-based handling of interoperable RDF data and dynamically generated data required for work with chemical databases. This is achieved by completely translating the incoming SPARQL queries into SQL queries enriched with stored procedure calls, which are then executed by a high-performance relational database.

This translation is performed by the SPARQL engine (displayed in the frontend section of Fig. 1). The dataset is stored in a PostgreSQL database in an optimized relational schema that allows high-performance querying. To represent the relational data in a given dataset as RDF, we created an *RDF/SQL mapping* that describes conversion of individual rows of tables in the relational schema into equivalent sets of RDF triples. This mapping is used to translate the incoming SPARQL queries into SQL queries that generate SPARQL-compatible query results. This mapping method is generally similar to the Linked Data Views in OpenLink Virtuoso [12].

Major differences between SPARQL and SQL functions and data types are addressed by using the custom PostgreSQL extension pgSPARQL (placed in the backend section in Fig. 1), which re-implements the SPARQL-specific behavior and functions in PostgreSQL.

The SPARQL engine is written in Java as a standard web application for Apache Tomcat. The frontend supports SPARQL 1.1 protocol for query submission. After the SPARQL query is translated into SQL and executed by PostgreSQL, the results are repacked to a user-selected standard query result format (XML, JSON, CSV or TSV [13–15]) and sent back to the client.

### Procedure call extension

Currently, there is no standardized way to represent a procedure call in SPARQL. Although a straight-forward syntactic extension for procedure calls could be implemented, it would make the service non-interoperable with existing standard-compliant services. Thus, we chose to adopt an approach from our earlier work [16]. We encode the procedure calls as SPARQL patterns that do not differ syntactically from regular query patterns, but are recognized by the SPARQL service upon processing and handled in a special way.

The procedure call pattern is a standard triple pattern with subject, predicate and object. The engine uses the predicate part of the pattern to identify the stored procedure to be called—a special IRI contained in the predicate triggers translation of the entire pattern into the corresponding SQL procedure call. Procedure call arguments are represented by a blank node contained in the pattern as an object; individual properties of the blank node represent individual named arguments of the procedure. This allows the user to specify parameters in any order, or omit them and use the default parameter values. Results of the procedure call are bound to the RDF node specified in the pattern as a subject, either directly if the returned result is not structured or as blank nodes with properties if the result is structured.

### Structure search using SPARQL

We mapped the high-performance substructure and similarity search SQL procedures from Sachem to corresponding SPARQL procedure calls. These are identified by the predicates sachem:substructureSearch and sachem:similaritySearch, respectively.

The procedure calls take several arguments. The main argument is sachem:query, which specifies either SMILES or MDL description of the queried molecule structure. A comprehensive list of other search arguments is provided in Table 1.

**Table 1:** Parameters of the chemical structure query executed via the SPARQL procedure call

Parameter name	Description and values
*Common parameters*	
sachem:query	Query molecule structure, formatted as SMILES or MDL
sachem:topn	Maximum number of results to return
*Substructure search parameters*	
sachem:searchMode	Chooses between exact structure and substructure search, values:
	sachem:substructureSearch
	sachem:exactSearch
sachem:tautomerMode	Tautomer handling, accepted values:
	sachem:ignoreTautomers (do not consider tautomerism)
	sachem:inchiTautomers (use InChI-based algorithm [17] for tautomer matching)
sachem:chargeMode	Selects coalescing of unspecified charge values in query:
	sachem:defaultChargeAsAny (unspecified charge is wildcard)
	sachem:defaultChargeAsZero (unspecified charge matches only uncharged atoms)
	sachem:ignoreCharges (ignores all charge annotations)
sachem:isotopeMode	Selects coalescing of unspecified isotope values in query:
	sachem:defaultIsotopeAsStandard (unspecified isotope matches only the standard isotope)
	sachem:defaultIsotopeAsAny (unspecified isotope is wildcard)
	sachem:ignoreIsotopes (ignore all isotope annotations)
sachem:stereoMode	Handling of stereochemistry:
	sachem:strictStereo (remove results with conflicting stereochemistry information)
	sachem:ignoreStereo (ignore all stereochemistry annotations)
*Similarity search parameters*	
sachem:cutoff	Minimum similarity score of returned results in range 0–1, defaults to 0.8

Using this framework, an example procedure call that finds all compounds that contain any tautomeric form of adenine as a substructure can be encoded to pattern as follows: 
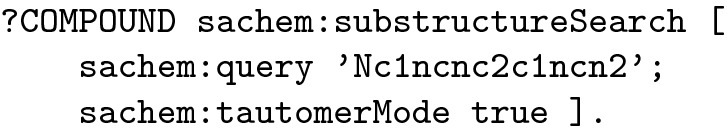


When included in a query that queries the ChEMBL dataset, the pattern will bind the variable COMPOUND to IRIs of the matching compounds (the result in CSV format is shortened for brevity): 
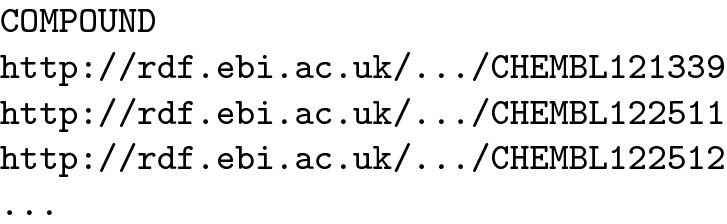


The results of the similarity search are structured values, encoded as blank RDF nodes with the properties sachem:compound and sachem:score, which represent the identifier of the retrieved compound and its similarity score, respectively. An example pattern that executes a search for compounds similar to adenine can be encoded as follows: 
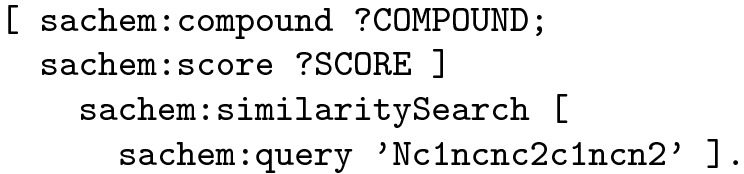


If included in the query, the similarity search results will be mapped to the two corresponding variables: 
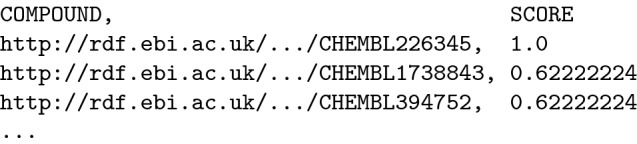


The returned similarity score is based on Jaccard similarity [18] of Morgan-style connectivity fingerprints [19] with a radius of up to 5.

### IDSM service dataset support

The service can provide chemical search functionality across datasets that have been indexed by the internal Sachem cartridge. IDSM currently includes the well-established public databases DrugBank [20], ChEBI [21], ChEMBL [22] and PubChem [23], thus providing fast search in more than 100 million total compounds.

Maintenance of the search indexes is crucial for the service to provide a lasting, reliable source of accurate search results. The IDSM service therefore aims to provide indexed data that is as up-to-date as possible. This is currently ensured by running automated nightly checks and updates of all datasets.

## Results

A running instance of the interoperable structure search service is accessible at the SPARQL endpoint https://idsm.elixir-czech.cz/sparql/endpoint/<database>, where ${\texttt {<database>}}$ comprises pubchem, drugbank, chebi and chembl.

We present two use cases: (1) a single-purpose chemical structure search application called ‘Sachem GUI,’ which serves as an example of using an interoperable chemical search in a web application, and (2) the general application ‘SPARQL GUI,’ which serves as a tool for constructing and running heterogeneous, federated queries that employ the service.

### Use case: chemical search in web applications

The possibility to obtain JSON-formatted output from the SPARQL interface makes the interoperable search functionality readily available for use in web applications. Public availability of this chemical information may be beneficial for construction of various domain-specific search tools and database access utilities atop the IDSM service.

We demonstrate this functionality in the online search tool ‘Sachem GUI,’ available at https://idsm.elixir-czech.cz/sachem. Sachem GUI represents the simplest possible JavaScript wrapper over the Sachem search functionality. It provides a user-friendly molecule-drawing interface for input of chemical structure queries (the JavaScript implementation of the drawing interface is based on the EPAM Ketcher tool^1^). After users draw a query, they may choose several other search parameters and hit the search button. Queries are then evaluated online by the IDSM service, and results are displayed as molecule images with metadata.

The source code of this application is intended as a starting point for development of more complicated search applications. For example, it could be easily adapted to allow the user to set additional search parameters and display the results of more complicated search queries, such as interactions with proteins (as shown in Sect. 3.3).

### Use case: heterogeneous chemical search

Examples of the various functionalities of federated queries across interoperable databases are presented in an interactive, modifiable form in the SPARQL GUI interface, available at https://idsm.elixir-czech.cz/sparql.

Technically, SPARQL GUI is a universal SPARQL interface enriched with IDSM-specific functionalities, which mainly comprise improved display of chemical results, available examples, and search API documentation. The JavaScript implementation of SPARQL GUI is based on YASGUI, which is a stand-alone SPARQL frontend.^2^ Queries tested in SPARQL GUI can be easily transferred to other software (e. g. web applications based on the first use case) or used to gather data programmatically.

### Heterogeneous query example

Of the examples available in SPARQL GUI, we selected protein–small molecule interactions to demonstrate the intended usage of the service.

It is generally straightforward to search for proteins that have a known interaction or measured activity with a small molecule—protein databases are already referenced by chemical databases that contain measured activities. This is also true for ChEMBL [22], which links chemical information to UniProt [24] references *via* a chain of activities, assays, targets and target components that ends in chembl:UniprotRef.

A related and potentially desirable search would be to query the activities of proteins with compounds that contain a certain molecular core bearing arbitrary additional ligands. For example, the user might be interested in protein activities with any existing derivatives of morphine. Such a query is, however, not directly executable using current database interfaces. To run it, the user would first have to employ a simple substructure search tool (either the ChEMBL online search or a self-hosted substructure search) to extract a potentially long list of morphine-containing ChEMBL IDs [22], which could then be used to construct a large query to obtain the protein information from UniProt.

**Fig. 2:**
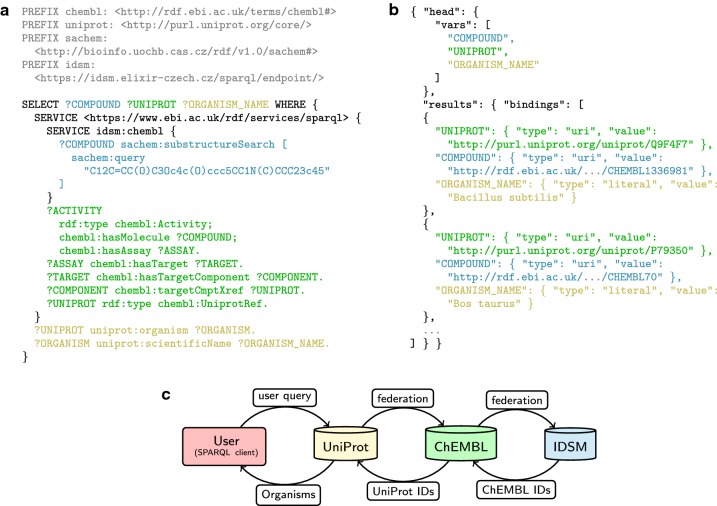
**a** An example of a federated SPARQL query that connects assay results of morphine derivatives to corresponding organism names. **b** Response to the same query in JSON format (shortened for brevity). **c** Schematic view of the distributed query processing. Colors match the execution place of the corresponding query parts, and the data source of the JSON response entries

With our interoperable chemical search service, this query can be run without any client-side overhead. Instead of listing the ChEMBL identifiers manually in the query, the user can employ a federated query that transparently retrieves the list of desired compound identifiers from the IDSM SPARQL endpoint. An example implementation of such a query is illustrated in Fig. 2, along with a schema of its distributed processing. To complete the example, the displayed query code connects the morphine derivatives with additional UniProt content to obtain more metadata, including the names of the corresponding organisms.

## Discussion

Representation of chemistry-related structure search results in the context of semantic search creates a new source of data linkages. The ability to query for structural similarity or sub- and super-structures of molecules enables retrieval of precisely specified chemical relations between identifiers of molecules, which can be transparently and transitively extended to any molecule-referencing data.

The new links can be used to alleviate some archetypal sources of trouble in chemical search, including the problems of annotation granularity and canonization.

Metadata about chemical compounds (e. g. assay results) are typically present only for a single variant of a molecule in a database, but may be valid for multiple different tautomeric structures or even some derivatives. If the user searches for a connection between two annotations (e. g. assay results and vendor availability) using only the exact molecule identifiers, this connection may be missed if the annotations were unintentionally placed with related (in this case, chemically identical) but differently identified molecules. The presented service allows users to enrich the search by including annotations from small similarity- or structure-defined ‘neighborhoods’ of molecules, thus effectively avoiding this problem in a wide variety of search scenarios.

This approach can be viewed as complementary to the more common canonization policies that are enforced in chemical databases [25, 26]. From a search perspective, both approaches help reduce the rate of false negatives by connecting larger amounts of available information. At the same time, they can potentially increase the rate of false positives. Manual annotation of canonized structure adds bias and maintenance problems derived from the human factor. Automatically produced links, on the other hand, may not have a chemically relevant interpretation and are biased by factors such as the similarity indexing method used.

## Conclusion

We have described a new service that delivers chemical structure search capabilities to searches in interoperable RDF databases. This innovation can be used to extend the rich heterogeneous search possibilities of current RDF databases by including automatically provided substructure and similarity relations between chemical identifiers in the search. We have demonstrated that this extension enables facile discovery of new, meaningful connections in data from multiple databases, and alleviates the impact of common canonization and curation problems in chemical databases.

The SPARQL engine is the main innovative component of the service. Although it is currently used only to provide an interoperable interface for Sachem, it is designed as a generic tool. Using the SPARQL engine, the same approach of improving dataset interoperability also can be applied to other areas of cheminformatics and bioinformatics, providing searches of metabolic pathways, biopolymer sequences, full-text metadata, etc.

The presented service is free, publicly available at https://idsm.elixir-czech.cz and accessible through a SPARQL-compatible machine interface. The service includes two demonstration user interfaces that can be used to manually test the service viability and as a basis for implementing the provided chemical search in new applications.

## Data Availability

All datasets used are publicly accessible the respective databases. The service is public and freely available.

## Footnotes

1 http://lifescience.opensource.epam.com/ketcher/.

2 https://github.com/OpenTriply/YASGUI.
