# Correction to: Interoperable chemical structure search service

**DOI:** 10.1186/s13321-020-0418-8

**Published:** 2020-02-13

**Authors:** Miroslav Kratochvíl, Jiří Vondrášek, Jakub Galgonek

**Affiliations:** 1 Institute of Organic Chemistry and Biochemistry of the CAS, Flemingovo náměstí 2, 166 10 Prague 6, Czech Republic; 2 Department of Software Engineering, Faculty of Mathematics and Physics, Charles University, Malostranské náměstí 25, 118 00 Prague 1, Czech Republic

## Correction to: J Cheminform (2019) 11:45 10.1186/s13321-019-0367-2

It was highlighted that the original article [1] contained an error in the last paragraph of the section ‘Structure search using SPARQL’, specifically in the radius of the used fingerprint. This Correction article shows the incorrect and correct paragraph of this section.

**Incorrect**

The returned similarity score is based on Jaccard similarity [18] of Morgan-style connectivity fingerprints [19] with a radius of up to 5.

**Correct**

The returned similarity score is based on Jaccard similarity [18] of Morgan-style connectivity fingerprints [19] with a radius of up to 3.
